# A Novel Splice Site Mutation of the ATM Gene Associated with Ataxia Telangiectasia

**Published:** 2018

**Authors:** Kolsoum SAEIDI, Nasrollah SALEH GOHARI, Seyed Ebrahim MANSOURI NEJAD

**Affiliations:** 1 Neurology Research Center, Kerman University of Medical Sciences, Kerman, Iran; 2 Department of Medical Genetics, School of Medicine, Kerman University of Medical Sciences, Kerman, Iran; 3 Department of Clinical Genetics, VU University Medical Center, Amsterdam, The Netherlands; 4 Herbal and Traditional Medicine Research Center, Kerman University of Medical Sciences, Kerman, Iran; 5 Department of Pediatrics, School of Medicine, Kerman University of Medical Sciences, Kerman, Iran

**Keywords:** Ataxia telangiectasia, Cerebellar ataxia, Splice site, New mutation, Autosomal recessive

## Abstract

**Objectives:**

Ataxia telangiectasia (AT) is a rare autosomal recessive disorder caused by mutation in the Ataxia telangiectasia mutated (ATM) gene. This disorder is characterized by progressive cerebellar ataxia, telangiectasia, immunodeficiency and a predisposition to leukemia/lymphoma. In this study, we investigated a family with a new mutation in ATM, confirmed by molecular genetic test.

**Materials&Methods:**

Four members of a family including a symptomatic AT patient, his parents and sibling were examined for ATM gene defects at Kerman University Hospital, Kerman, Iran in 2016. DNA was extracted from peripheral leukocytes and the coding regions and exon-intron boundaries of ATM gene were amplified by next-generation sequencing technique. The identified mutation was tested in all members of the family.

**Results:**

Molecular analyses identified a homozygous T to G substitution in c.7308-6 position resulting in a novel acceptor splice site in intron 49 of the ATM gene in the index patient. Parents and sibling of the proband were heterozygous for the same mutation.

**Conclusion:**

The variant c.7308-6T>G is predicted to be pathogenic due to impaired splice site causing exon skipping. This newly found frameshift mutation cosegregated as an autosomal recessive trait as expected for Ataxia telangiectasia syndrome.

## Introduction

Autosomal recessive cerebellar ataxias (ARCAs) are a heterogeneous group of neurological disorders, characterized by degeneration or abnormal development of cerebellum and spinal cord. In most patients, onset age is before 30 year. The most frequent ARCAs in Caucasian population is Friedreich ataxia, ataxia-telangiectasia (AT) and early onset cerebellar ataxia with retained tendon reflexes. Gait ataxia is the common manifestation of these disorders. Other common findings include nystagmus, dysarthria, and dysmetria. Brain imaging often shows cerebellar atrophy or hypoplasia. Age of onset varies widely but is frequently in childhood. Intellectual disability, peripheral neuropathy, and retinal abnormalities may also occur (1). 

Clinical diagnosis is confirmed by magnetic resonance imaging (MRI), electrophysiological examination, and mutation analysis. Recently, molecular genetic has been a powerful approach in investigating inherited ataxias (2).

ARCAs have been divided to (1, 3): 1) Congenital ataxias which include Joubert syndrome (Dysgenesis or agenesis of the vermis and presence of molar tooth sign) and Cayman cerebellar ataxia (Hypotonia from birth, non-progressive truncal and limb ataxia). 2) Metabolic ataxias which include ataxia with isolated vitamin E deficiency (Very low plasma level of vitamin E and normal lipid profile), Abetalipoproteinemia (apolipoproteins B deficiency, multiple fat-soluble vitamin deficiency and abnormal lipidogram), Cerebrotendinous xanthomatosis (Bile acid biosynthesis defect, large deposit of cholesterol and xanthomas) and Refsum disease (Retinitis pigmentosa, deafness, increased phytanic acid level in blood and abnormal blood lipids). 3) Degenerative and progressive ARCAs which include Friedreich ataxia (Ataxia with scoliosis, foot deformity, cardiac symptoms, defective mitochondrial proteins, absence of telangiectasia and normal α-fetoprotein), Mitochondrial recessive ataxia syndrome (Mitochondrial DNA replicative polymerase defect, Infantile onset spinocerebellar ataxia (Mostly Finish heritage disease with hypotonia and vision and hearing problems), Charlevoix-Saguenay spastic ataxia (Early onset, progressive spastic ataxia of all limbs with paraplegia, increased tendon reflexes, progressive distal wasting, atrophy of the superior cerebellar vermis and Retinal hypermyelinated), Marinesco-Sjögren syndrome (Ataxia with congenital cataract, elevation of serum creatine kinase activity, Hypergonadotropic hypogonadism, facial dysmorphism and atrophy of the vermis), Early onset cerebellar ataxia with retained tendon reflexes (Early onset cerebellar ataxia with preservation of deep tendon reflexes), Coenzyme Q10 deficiency with cerebellar ataxia (Reduced levels of coenzyme Q10 in muscle biopsies), and Posterior column ataxia and retinitis pigmentosa (Posterior column ataxia and retinitis pigmentosa). 4) DNA repair defects which include ataxia telangectasia-like disorder (Different from AT based on the genetic data), ataxia with oculomotor apraxia-1 (Limb dysmetria, hypoalbuminemia and hypercholesterolemia), ataxia with oculomotor apraxia-2 (Dystonic posturing with walking and elevated values of gamma-globulin, and creatin kinase), spinocerebellar ataxia with axonal neuropathy (Ataxia with peripheral axonal motor and sensory neuropathy, distal muscular atrophy, and pes cavus), Xeroderma pigmentosum (Ataxia with skin photosensitivity, early onset skin cancers, photophobia, conjunctivitis, keratitis, ectropion and entropion), and AT (Progressive ataxia, with oculocutaneous telangiectasia, and variable immunodeficiency). 

AT is also characterized by sinopulmonary infections, radiosensitivity, early aging, chromosomal instability and a predisposition to leukemia/lymphoma. It is often misdiagnosed in early childhood, before the development of a full clinical picture. The first signs of the disease are movement disorders with different severity. The incidence of AT is 1/40000-100000 cases, but in several populations, the incidence is considerably higher due to founder mutations (4-6). 

The initial clinical description of AT disease is reported earlier (7). Three adolescent Czech siblings were described with progressive chorea, dystonia, and ocular telangiectasia. Later, a 9-yr-old child was described with progressive cerebellar ataxia and bilateral oculocutaneous telangiectasia in details (8). The term ataxia telangiectasia was introduced in 1958 and recognized as autosomal recessive mode of inheritance for the disease (9). The disease is sometimes referred to as Boder-Sedgwick syndrome. Different groups have reported case series of AT patients (9-14). 

AT is caused by mutations in the Ataxia telangiectasia mutated (ATM) gene, consisting of 66 exons, in chromosome 11 (11q22.3). A 370 kDa protein encoded by ATM gene is an important checkpoint kinase which is one of the most important controllers of cell cycle checkpoint signaling pathways required for cell response to DNA damage and for genome stability. Therefore, nuclear genomic instability resulting from loss of this function is regarded as a major mechanism underlying the pathology of AT (5, 15). 

In contrast to ARCAs, autosomal dominant cerebellar ataxias are characterized by late onset spinocerebellar ataxias and episodic ataxias (16).

In this study, we investigated a family with a new mutation in ATM, confirmed by molecular genetic test.

## Materials & Methods

**Fig 1:**
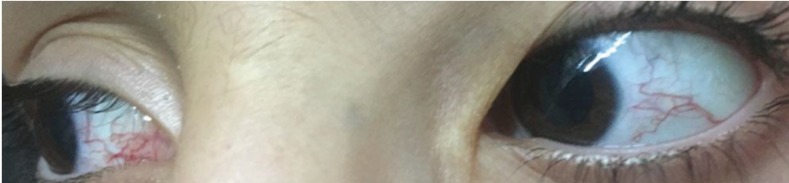
Ocular telangiectasia in patient

In 2016, an 8-yr-old boy born of a consanguineous marriage was admitted to the Kerman University Hospital, Kerman, Iran with the complaints of generalized weakness and difficulty to walk. The patient had one healthy younger sister of 5 yr old. His birth history was uneventful and onset of disease occurred about 2 yr of age with loss of balance. The progression of the ataxia increasingly developed beyond age 5 year. He had ataxic gait, abnormal head movements, chorea, myoclonus, slurred speech, oculomotor apraxia, neuropathy, intention tremor, and hand incoordination. He had drooling and frequent experiences of aspiration because swallowing was not well coordinated. He suffered from frequent respiratory tract infections due to low levels of IgG, IgA, and IgG subclasses. We found congestions in both eyeballs (Figure 1) which appeared between 5 and 6 yr of age and nystagmus. He showed also Café au lait spots. His intelligence was normal and he was cooperative. Writing was affected by age 7 year. Deep tendon reflexes were slight but present. 

A genetic counselor explained the objectives and aims of the study to participants. Written informed consents were obtained from subjects. 

The 5 ml whole blood from subjects’ brachial vein in tubes containing 200 µl EDTA was collected to detect any ATM gene mutations. Genomic DNA was isolated from leukocytes of the whole blood using salt-saturation method (17). In AT work-up, the DNA sample of an 8-yr-old affected member of the family was screened for the gene defects using next-generation sequencing technique. The sequencing processes were performed on Illumina Hiseq 4000 platform. Sequence reads were analyzed using BWA –GATK (18, 19). The other family members were screened for the identified mutation by PCR and DNA sequencing methods. To accomplish this, a 572 bp DNA fragment of the ATM gene including intron 49, exon 50 and exon-intron boundaries were amplified using a forward primer 

(5'-AGTGTAAGCAGAGGTGTAAGTTA-3') and a reverse primer 

(5'-CACTGGACCAAGTGCTAGGAATA-3'). The PCR reaction was performed using the following condition: 30 cycles of denaturation at 94 °C for 45 sec, annealing at the optimal temperature of each primer for 30 sec and extension at 72 °C for 30 sec. PCR products were checked on 1% agarose gel and taken through an ABI automated DNA sequencer (Model: 3730XL).

## Results

Complete blood count, lipid profile, LFT, chest X-ray, serum electrolytes, pyruvate, ammonia, lactate, levels of vitamins B12 and E were normal in the proband, he had low level of IgG, IgA, and IgG subclasses, ultrasonogram of whole abdomen and ECG were normal. Serum α-fetoprotein was high and MRI showed cerebellar atrophy. Four members of family including a symptomatic AT patient, his parents and sibling were examined for ATM gene defects. 

**Fig 2:**
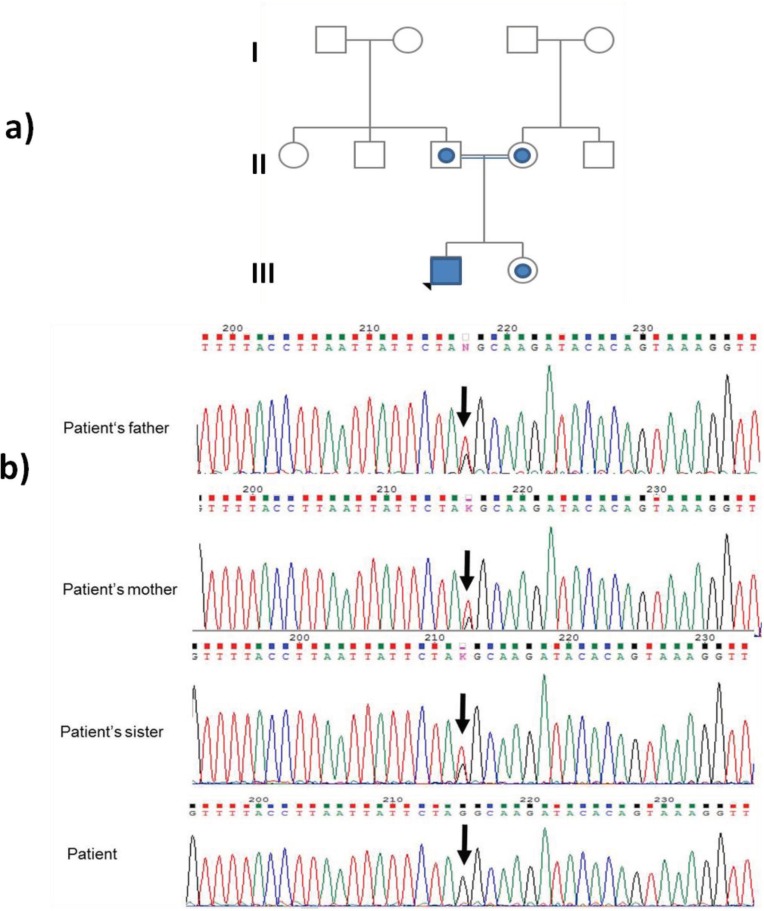
a) Pedigree of the family with AT. The circle indicates female, the square indicates male, and the filled square indicates the affected individual. b) The homozygous mutation detected in the ATM gene in the patients and heterozygous mutation detected in his parents and sibling. Nucleotide variation is indicated by an arrow.

**Fig 3:**
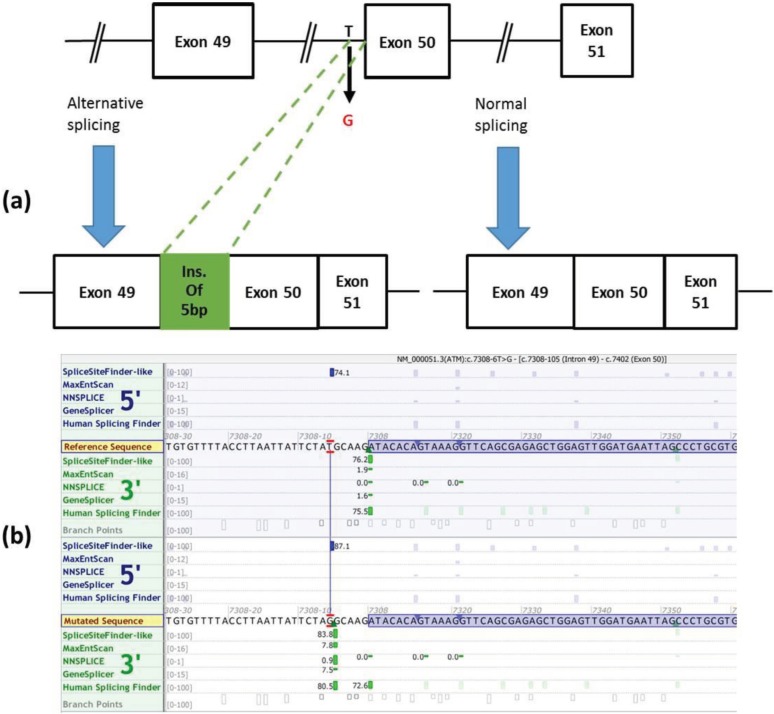
(a) Ideogram of detected splicing consequences of the c.7308-6 T>G mutation. (b) c.7308-6T>G mutation has moved initial acceptor site by 5 bp upstream from its normal position. The window in (b) displays the reference and mutated sequences and green vertical bars for 3' (acceptor) sites. The height of each bar is proportional to the maximum possible score.

The screening of the ATM gene in patient revealed T to G substitution in c.7308-6 position. This intronic variant appeared in a homozygous state. The asymptomatic sister of the index patient and his parents were heterozygous for this mutation (Figure 2). Using In Silico Tools for splicing defect prediction (20) and Alamut® Visual software version 2.8, the variant c.7308-6T>G in intron 49 is predicted to be pathogenic due to impaired splice site. Based on the In Silico prediction tools, c.7308-6T>G mutation has moved initial acceptor site by 5 bp upstream from its normal position. This causes an abnormal splicing process by activation of a new cryptic splice acceptor site. The aberrant splicing causes exon skipping (Figure 3). 

**Discussion**

AT is a complex multisystem disorder caused by a mutation in the ATM gene. The ATM gene encodes the protein kinase ATM which expressed is mainly in the nucleus of lymphocytes, fibroblasts, germ cells, and neurons. This protein is the key regulator of DNA damage response (21, 22). The AT gene was mapped to chromosome 11q22.3 (23).

ATM gene was mutated in AT (24). Since then, several mutations in ATM gene including truncating mutations, which result in the total absence of ATM kinase activity, and missense or splice site mutations, leading to decreased kinase activity have been described (25-29). Up to date, more than 400 mutations in the ATM gene have been described in AT patients (6). 

We found a new mutation (c.7308-6T>G) in an 8-yr-old boy with AT who was initially diagnosed with loss of balance. Early diagnosis was delayed due to the rarity of the disease and also lack of obvious clinical symptoms which started at 5 yr of age. Several other neurologic and rare disorders should be taken into account by physicians when aiming for the AT diagnosis (2). Some lab tests such as gene sequencing as well as the clinical features of the disease would assist in differential diagnosis (2). The diagnosis was established on the basis of clinical features such as progressive cerebellar dysfunction, gait and truncal ataxia, telangiectasia, head tilting, impaired eye movement, immunodeficiency and recurrent sinopulmonary infections, dysarthria, slight deep tendon reflexes, elevated serum α-fetoprotein and normal height and intelligence. 

We conclude that T to G substitution in c.7308-6 position modifies pattern of splicing leading to a five-base insertion in the transcript, which causes exon skipping. Splicing site prediction tools gave the highest sensitivity scores of 80.5%- 90% for initial acceptor site changing. This skipped exon is located in FAT domain of protein. Of note, the importance of the FAT domain, as a structural scaffold or as a protein-binding domain, has been reported (30). Exon skipping is a very strong evidence (PVS1) of pathogenicity according to the ACMG (American College of Medical Genetics and Genomics) guidelines (31). Moreover, this new mutation also fulfil the supporting criteria pp3 (deleterious effect on the gene or gene product such as conservation has been supported by various computational evidences) and pp4 (patient’s phenotype is highly specific for a disease with a single genetic etiology) as well as two moderate criteria PM1 (Located in a critical and well-established functional domain e.g. active site of an enzyme) and PM2 (Absent from controls in Exome Sequencing Project, 1000 Genomes or ExAC) (31).

Therefore, enough lines of evidence are invoked to classify this variant as a pathogenic mutation based on the ACMG variant classification guidelines. 

**In Conclusion, **the variant c.7308-6T>G has not been reported previously and is predicted to be pathogenic due to impaired splice site causing exon skipping. However, as a limitation, we did not have more cases to confirm this. Therefore, further investigation on the functional role and clinical impact of novel alteration are proposed.
